# Association between serum bicarbonate levels and 28-day in-hospital mortality in dialysis patients: a multicenter retrospective cohort study based on the eICU Collaborative Research Database

**DOI:** 10.3389/fmed.2025.1607191

**Published:** 2025-11-12

**Authors:** Die Wu, Yuemei Liu, Yueyun Zeng, Ridong Lai, Xinglin Chen, Dan Wu

**Affiliations:** 1 Department of Chinese Medicine and Anorectology, Fuyong People’s Hospital of Baoan District, Shenzhen, China; 2 Fuqiao Community Health Center, Fuyong People’s Hospital of Baoan District, Shenzhen, China; 3 Department of Epidemiology and Biostatistics, Empower U.X&Y Solutions Inc., Boston, MA, United States; 4 Department of Gynecology Ward 1, Gaozhou People’s Hospital, Maoming, China

**Keywords:** serum bicarbonate, dialysis, mortality, ICU, ESKD

## Abstract

**Background and aims:**

The relationship between serum bicarbonate levels and 28-day mortality in dialysis patients remains unclear. This study aimed to investigate the association between serum bicarbonate levels and short-term mortality in patients undergoing dialysis.

**Methods:**

This multicenter retrospective cohort study included 4,979 dialysis patients aged 18 years or older from the electronic Intensive Care Unit (eICU) Collaborative Research Database (2014–2015). Serum bicarbonate levels were measured within 24 h of ICU admission. A multivariate Cox regression model was applied to evaluate the association between serum bicarbonate levels and 28-day mortality.

**Results:**

A total of 4,979 patients were analyzed, with a median age of 63 years. Among them, 513 patients (10.3%) died within 28 days. A significant non-linear relationship was observed between serum bicarbonate levels and mortality. Using a two-segment linear regression model, the inflection point was determined to be 30 mmol/L (log-likelihood ratio test, *p* = 0.029). Below this threshold, serum bicarbonate was inversely associated with 28-day mortality (OR = 0.89, 95% CI: 0.86–0.92, *p* < 0.0001). Above the threshold, the association was not statistically significant (OR = 1.11, 95% CI: 0.97–1.28, *P* = 0.1278).

**Conclusion:**

Serum bicarbonate levels are non-linearly associated with 28-day mortality in dialysis patients. Levels below 30 mmol/L are linked to an increased risk of death. These findings need to be confirmed in future prospective studies.

## 1 Introduction

Patients with end-stage kidney disease (ESKD) rely on dialysis therapy to sustain life, yet, despite significant advances in technology, mortality in this population remains unacceptably high. Recent studies have shown that mortality risk in dialysis is influenced by several factors, including dialysis modality, adequacy, use of erythropoiesis-stimulating agents, nutritional status, and blood pressure control. The early post-initiation period of dialysis is associated with the highest mortality risk. For example, a large Korean cohort study reported a 19.3% overall mortality rate during a median follow-up of 66.2 months in patients receiving hemodialysis (1). Similar findings have been reported globally, with Bossola et al. further corroborating elevated short-term mortality following the initiation of dialysis (2). These data emphasize the need to identify early prognostic indicators in dialysis patients.

Serum bicarbonate is essential for homeostasis and plays a key role in maintaining acid-base balance. Abnormal bicarbonate levels are associated with poor outcomes in a variety of clinical settings. In dialysis populations, both low ( ≤ 17 mmol/L) and high ( > 27 mmol/L) bicarbonate levels have been associated with increased mortality, whereas levels above 22 mmol/L are generally linked to better survival (3). Dialysate bicarbonate concentration has also been shown to positively correlate with all-cause mortality, and fluctuations in bicarbonate levels may influence hemodynamic stability and prognosis (4). Despite these observations, the association between serum bicarbonate and short-term mortality in dialysis patients remains poorly defined. Most prior studies have emphasized long-term outcomes and report inconsistent threshold values, limiting their clinical utility (5–7). This inconsistency highlights the need for large-scale, standardized investigations to identify reliable cutoff points for guiding clinical decisions.

Based on the current literature, we hypothesized that both high and low serum bicarbonate levels are associated with increased risk of 28-day all-cause mortality in dialysis patients. To test this hypothesis, we conducted a multicenter retrospective cohort study using data from the eICU Collaborative Research Database, which includes detailed ICU data from 208 hospitals in the United States between 2014 and 2015. Our objective was to determine whether serum bicarbonate levels are independently associated with short-term mortality and to identify potential threshold values that may inform clinical management in critically ill dialysis patients.

## 2 Materials and methods

### 2.1 Data source

This retrospective cohort study was conducted using data from the electronic Intensive Care Unit Collaborative Research Database (eICU-CRD) (8). The eICU-CRD is a multicenter ICU database comprising over 200,000 admissions from 208 hospitals in the United States between 2014 and 2015 (8). Data were collected automatically via the Philips Healthcare eICU program and made available for research purposes. One of the authors (Xinglin Chen) was granted access and performed data extraction (certification number: 40859994). The use of eICU-CRD has been approved by the Institutional Review Board of the Massachusetts Institute of Technology (Cambridge, MA, United States) and has been widely applied in observational research (9–11). As this study was retrospective and based on de-identified data, informed consent was not required. All procedures complied with relevant ethical regulations and data-use agreements.

### 2.2 Study population

**FIGURE 1:**
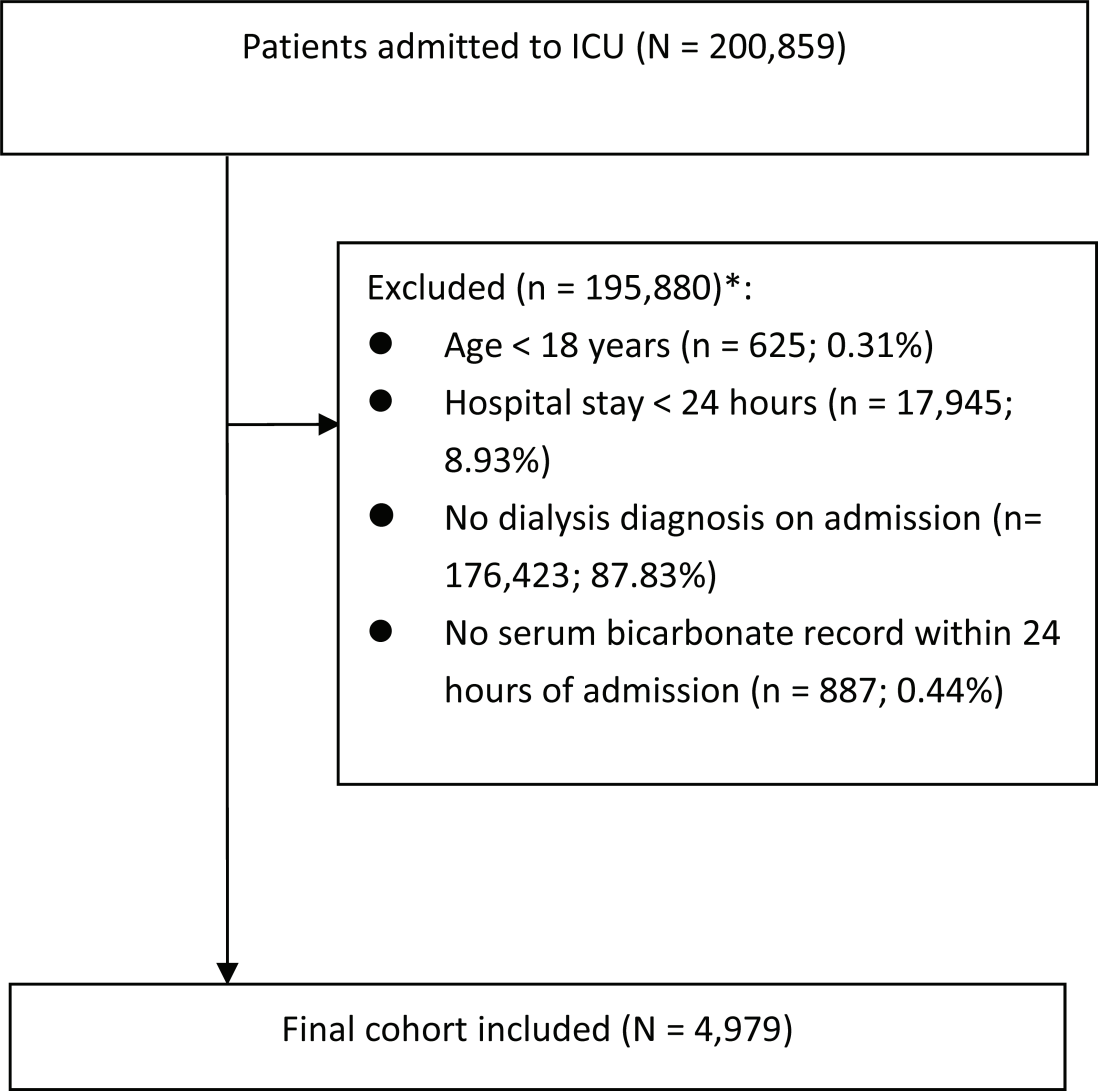
Flow chart of study population. *Subjects selected after each step. ICU, intensive care unit.

The initial cohort consisted of 200,859 patients. After applying exclusion criteria, 4,979 patients were included in the final analysis. Exclusion criteria were as follows: (1) age under 18 years; (2) hospital stay shorter than 24 h; (3) absence of dialysis initiation within 24 h of admission; and (4) no serum bicarbonate measurement within the first 24 h after admission. A detailed study flowchart is shown in Figure 1.

### 2.3 Variables

The eICU database includes comprehensive patient data such as demographics, physiological parameters, ICD-9-CM diagnoses, and laboratory results. All variables were obtained within the first 24 h of admission.

The primary exposure was serum bicarbonate (mmol/L), defined as the initial measurement within the first 24 h of ICU admission. We adopted this window to enhance completeness and cross-site comparability in the eICU database, where early labs have the lowest missingness. Covariates were selected based on clinical relevance and prior literature (12, 13), and included age, gender, ethnicity, body mass index (BMI, kg/m^2^), and the Sequential Organ Failure Assessment (SOFA) score. Laboratory parameters included serum creatinine, sodium, potassium, calcium, and white blood cell (WBC) count. Additionally, clinical interventions and comorbidities included intubation status, mechanical ventilation, vasopressor dose, FiO_2_, presence of chronic heart failure (CHF), chronic obstructive pulmonary disease (COPD), and diabetes mellitus (DM), all determined from the patient’s medical history.

### 2.4 Outcomes

The primary outcome was 28-day in-hospital mortality, defined as death occurring within 28 days after admission.

### 2.5 Statistical analysis

Descriptive statistics were used to characterize the study population. Continuous variables were summarized as mean ± standard deviation for normally distributed data or median with interquartile range (IQR) for skewed data. Categorical variables were expressed as counts and percentages. Comparisons across serum bicarbonate tertiles were conducted using one-way analysis of variance (ANOVA) for normally distributed continuous variables, the Kruskal-Wallis test for skewed data, and the chi-square test for categorical variables. To avoid the loss of statistical power and bias associated with listwise deletion, we employed multiple imputation (MI) to estimate missing values.

A generalized additive model (GAM) was applied to explore the non-linear association between serum bicarbonate levels and 28-day mortality. Univariate and multivariate binary logistic regression models were used to evaluate this association. Two adjusted models were constructed to control for potential confounders. Covariates included age, gender, ethnicity, BMI, SOFA score, serum creatinine, sodium, potassium, calcium, white blood cell count, intubation status, mechanical ventilation, CHF, COPD, and DM. Odds ratios (ORs) with 95% confidence intervals (CIs) were reported. To ascertain the robustness of the results, sensitivity analyses were conducted. All statistical analyses were carried out using R software version 4.0.0 and Empower Stats (X&Y Solutions, Inc., Boston, MA). A two-sided *p*-value of less than 0.05 was considered statistically significant.

## 3 Results

### 3.1 Baseline characteristics

Data from 4,979 patients were analyzed. The median age was 63 years [interquartile range (IQR) 53–72], and 2,760 (55.43%) were female. A significant age difference was observed across serum bicarbonate tertiles (*p* < 0.001), whereas gender (*p* = 0.988) and ethnicity distribution (*p* = 0.061) did not differ significantly. Body mass index (BMI) also showed a statistically significant association with serum bicarbonate levels (*p* = 0.024). Additional significant differences among tertiles were observed in serum creatinine, calcium, potassium, sodium, white blood cell count, and intubation status (*p* < 0.001). The use of mechanical ventilation differed significantly across serum bicarbonate groups (*p* = 0.048), as did FiO_2_ (%) (*p* = 0.003) and vasopressor use in the first 24 h, specifically in the “no-use” category (*p* = 0.048). Similarly, chronic obstructive pulmonary disease (COPD) and chronic heart failure (CHF) were associated with varying bicarbonate levels. Full baseline characteristics and the associations between serum bicarbonate levels and clinical variables are provided in Table 1.

**TABLE 1:** Baseline characteristics of participants (*n* = 4,979).

Characteristics	Bicarbonate (mmol/L)			
	Tertile 1	Tertile 2	Tertile 3	*P*-value
	4–21	22–27	28–52	
	1,565	1,371	2,043	
Age (years)	59.63 ± 14.56	61.25 ± 14.33	63.01 ± 14.17	< 0.001
Gender, n (%)				0.988
Male	700 (44.73)	610 (44.49)	909 (44.49)	
Female	865 (55.27)	761 (55.51)	1,134 (55.51)	
Ethnicity, n (%)				0.061
Caucasian	868 (55.68)	734 (53.81)	1,145 (56.18)	
African American	469 (30.08)	402 (29.47)	539 (26.45)	
Hispanic	62 (3.98)	77 (5.65)	122 (5.99)	
Asian	89 (5.71)	85 (6.23)	141 (6.92)	
Native American	34 (2.18)	38 (2.79)	54 (2.65)	
Other/Unknown	37 (2.37)	28 (2.05)	37 (1.82)	
BMI (kg/m^2^)	28.32 ± 7.63	29.13 ± 8.52	28.85 ± 8.19	0.024
Serum creatinine (mg/dL)	6.14 (4.27–8.90)	5.47 (3.90–7.73)	4.82 (3.41–6.76)	< 0.001
SOFA score	5.95 ± 2.66	5.66 ± 2.48	5.15 ± 2.30	< 0.001
Sodium (mmol/L)	135.77 ± 4.92	136.40 ± 4.12	137.12 ± 3.70	< 0.001
Potassium (mmol/L)	4.70 ± 1.00	4.49 ± 0.84	4.34 ± 0.80	< 0.001
WBC (10^∧^9/L)	11.07 (7.80–16.40)	10.10 (7.10–14.40)	8.84 (6.40–12.40)	< 0.001
Calcium (mg/dL)	8.30 ± 0.96	8.49 ± 0.80	8.58 ± 0.80	< 0.001
Intubated				< 0.001
No	1,285 (82.11)	1,156 (84.32)	1,812 (88.69)	
Yes	280 (17.89)	215 (15.68)	231 (11.31)	
Mechanical ventilation use				0.048
No	1,104 (70.54)	975 (71.12)	1,511 (73.96)	
Yes	461 (29.46)	396 (28.88)	532 (26.04)	
COPD, n (%)				0.013
No	1,485 (94.89)	1,284 (93.65)	1,889 (92.46)	
Yes	80 (5.11)	87 (6.35)	154 (7.54)	
CHF, n (%)				< 0.001
No	1,391 (88.88)	1,190 (86.80)	1,684 (82.43)	
Yes	174 (11.12)	181 (13.20)	359 (17.57)	
DM, n (%)				0.601
No	1,239 (79.17)	1,076 (78.48)	1,589 (77.78)	
Yes	326 (20.83)	295 (21.52)	454 (22.22)	
**Vasopressor use (1st 24 h), n (%)**				
No	1,104 (70.54)	1,104 (70.54)	1,104 (70.54)	0.048
Yes	461 (29.46)	461 (29.46)	461 (29.46)	
FiO_2_(%)	49.95 ± 32.57	57.64 ± 168.06	45.27 ± 27.61	0.003
Time hospital 28-day	6.24(3.67–10.87)	5.99 (3.61–10.66)	5.54 (3.04–9.70)	< 0.001
Hospital 28-day mortality				< 0.001
No	1,325 (84.66)	1,236 (90.15)	1,905 (93.25)	
Yes	240 (15.34)	135 (9.85)	138 (6.75)	

Results in table: Mean ± SD, Median (Q1−Q3)/ n (%).BMI, body mass index; SOFA score, Sequential Organ Failure Assessment; WBC, white blood cell; COPD, chronic obstructive pulmonary disease; CHF, congestive heart failure; DM, diabetes mellitus; Vasopressor use (1st 24 h); FiO_2_.

Data are presented as mean ± standard deviation, median (Q1−Q3), or n (%) as appropriate. Among the 4,979 participants, missing data were observed in the following variables: race/ethnicity (*n* = 18, 0.36%), BMI (*n* = 198, 3.97%), serum calcium (*n* = 60, 1.20%), serum creatinine (*n* = 11, 0.22%), serum potassium (*n* = 8, 0.16%), and white blood cell count (*n* = 269, 5.40%), Vasopressor use (*n* = 9, 0.18%); FiO_2_ (*n* = 2932, 58%).

### 3.2 28 -Day mortality

The overall 28-day mortality rate was 10.30% [513 of 4,979 patients; 95% confidence interval (CI): 9.46–11.15%]. Mortality rates across bicarbonate tertiles were as follows: 15.34% in the lowest tertile (4–21 mmol/L), 9.85% in the middle tertile (22–27 mmol/L), and 6.75% in the highest tertile (28–52 mmol/L), as shown in Table 1.

### 3.3 Univariate analysis for 28-day mortality

Results of the univariate logistic regression are shown in Table 2. Serum bicarbonate level was significantly and inversely associated with 28-day mortality [odds ratio (OR) = 0.91, 95% CI: 0.89–0.93, *p* < 0.0001], with a clear dose-response relationship observed across tertiles. Compared with the lowest tertile, patients in the middle tertile had a 40% lower risk of mortality (OR = 0.60; 95% CI: 0.48–0.75; *p* < 0.0001), and those in the highest tertile had a 60% lower risk (OR = 0.40; 95% CI: 0.32–0.50; *p* < 0.0001). Additional factors independently associated with higher mortality included age, SOFA score, white blood cell count, intubation, mechanical ventilation, and vasopressor use in the first 24 h. Mechanical ventilation was strongly associated with higher 28-day mortality. In univariate analysis, ventilated patients had significantly greater odds of death compared with non-ventilated patients (OR = 2.56, 95% CI: 2.13–3.08, *p* < 0.0001). Intubation status was likewise associated with increased 28-day mortality. Patients who were intubated had higher odds of death than those not intubated (OR = 3.12, 95% CI: 2.54–3.84, *p* < 0.0001). SOFA score, as a proxy for illness severity, showed a positive and monotonic association with 28-day mortality. Each one-point increase in SOFA was linked to higher odds of death (OR = 1.32 per point, 95% CI: 1.28–1.37, *p* < 0.0001), and when categorized, patients in higher SOFA categories had substantially greater risk relative to the lowest category, consistent with a dose–response pattern. Interestingly, higher serum creatinine levels were associated with reduced mortality risk; patients in the highest serum creatinine tertile had a lower risk (OR 0.44, 95% CI: 0.35–0.56, *p* < 0.0001).

**TABLE 2:** The univariate analysis of 28-day mortality.

Exposure	Statistics	OR (95%CI)	*P-*value
Bicarbonate (mmol/L)	24.34 ± 4.53	0.91 (0.89, 0.93)	< 0.0001
**Bicarbonate (mmol/L) tertile**			
Tertile 1 (4–21)	1,565 (31.43)	Reference	
Tertile 2 (22–27)	1,371 (27.54)	0.60 (0.48, 0.75)	< 0.0001
Tertile 3 (28–52)	2,043 (41.03)	0.40 (0.32, 0.50)	< 0.0001
Age (years)	61.46 ± 14.41	1.02 (1.01, 1.03)	< 0.0001
**Gender, n (%)**			
Male	2,219 (44.57)	Reference	
Female	2,760 (55.43)	1.04 (0.87, 1.25)	0.6641
**Ethnicity**			
Caucasian	2,747 (55.37)	Reference	
African American	1,410 (28.42)	0.81 (0.66, 1.01)	0.0613
Hispanic	261 (5.26)	0.77 (0.49, 1.20)	0.2442
Asian	315 (6.35)	0.57 (0.36, 0.90)	0.0155
Native American	126 (2.54)	1.50 (0.92, 2.45)	0.1067
Other/Unknown	102 (2.06)	0.96 (0.51, 1.82)	0.9019
BMI (kg/m^2^)	28.76 ± 8.12	1.00 (0.99, 1.01)	0.8087
**Serum creatinine (mg/dL)**			
0.07–3.75	1,645 (33.11)	Reference	
3.76–7.63	1,665 (33.51)	0.65 (0.53, 0.81)	< 0.0001
7.64–36.8	1,658 (33.37)	0.44 (0.35, 0.56)	< 0.0001
SOFA score	5.54 ± 2.49	1.32 (1.28, 1.37)	< 0.0001
Sodium (mmol/L)	136.50 ± 4.27	1.02 (1.00, 1.04)	0.0882
Potassium (mmol/L)	4.50 ± 0.89	0.89 (0.80, 0.99)	0.0369
**WBC (10^∧^9/L) tertile**			
0.1–7	1,563 (33.18)	Reference	
7.1–14	1,577 (33.48)	1.62 (1.23, 2.13)	0.0006
14.1–179.3	1,570 (33.33)	3.34 (2.60, 4.29)	< 0.0001
Calcium (mg/dL)	8.47 ± 0.86	0.97 (0.87, 1.08)	0.5588
Intubated			
No	4,253 (85.42)	Reference	
Yes	726 (14.58)	3.12 (2.54, 3.84)	< 0.0001
**Mechanical ventilation use**			
No	3,590 (72.10)	Reference	
Yes	1,389 (27.90)	2.56 (2.13, 3.08)	< 0.0001
**COPD, n (%)**			
No	4,658 (93.55)	Reference	
Yes	321 (6.45)	1.03 (0.72, 1.49)	0.8604
**CHF, n (%)**			
No	4,265 (85.66)	Reference	
Yes	714 (14.34)	0.82 (0.62, 1.08)	0.1604
**DM, n (%)**			
No	3,904 (78.41)	Reference	
Yes	1,075 (21.59)	0.88 (0.70, 1.10)	0.2690
**Vasopressor use (1st 24 h), n (%)**			
No	4,933 (99.26)	Reference	
Yes	37 (0.74)	3.73 (1.83, 7.60)	0.0003
FiO_2_(%)	50.28 ± 92.42	1.00 (1.00, 1.00)	0.1922

Results in table: Mean ± SD/n (%); Median (Q1-Q3)/ n (%). BMI, body mass index; SOFA score, Sequential Organ Failure Assessment; WBC, white blood cell; COPD, chronic obstructive pulmonary disease; CHF, congestive heart failure; DM, diabetes mellitus, Vasopressor use (1st 24 h); FiO_2_.

### 3.4 Association between serum bicarbonate and 28-day mortality

In the unadjusted model, each 1 mmol/L increase in serum bicarbonate was associated with a lower mortality risk (OR = 0.91, 95% CI: 0.89–0.93, *p* < 0.00001). This inverse association persisted after multivariate adjustment. Patients in the middle and highest tertiles had significantly reduced mortality risk compared to those in the lowest tertile. Specifically, the OR was 0.50 (95% CI: 0.36–0.70, *p* < 0.0001) in the middle tertile and 0.38 (95% CI: 0.27–0.55, *p* < 0.00001) in the highest tertile (Table 3).

**TABLE 3:** The relationship between bicarbonate and 28-day mortality.

Outcome	Non-adjusted model (OR, 95%, *P*)	Adjusted model I (OR, 95%, *P)*	Adjusted model II (OR, 95%, *P*)
Bicarbonate (mmol/L)	0.91 (0.89, 0.93) < 0.0001	0.90 (0.88, 0.92) < 0.0001	0.90 (0.87, 0.93) < 0.0001
**Bicarbonate (mmol/L) tertile**			
Low	Reference	Reference	Reference
Middle	0.60 (0.48, 0.75) < 0.0001	0.58 (0.46, 0.73) < 0.0001	0.50 (0.36, 0.70) < 0.0001
High	0.40 (0.32, 0.50) < 0.0001	0.37 (0.29, 0.46) < 0.0001	0.38 (0.27, 0.55) < 0.0001

OR, odds ratio. Model I adjusted for Gender and Age (years). Model II adjusted for age (years), gender, ethnicity; BMI, body mass index; WBC, white blood cell; SOFA score, Sequential Organ Failure Assessment; COPD, chronic obstructive pulmonary disease; CHF, congestive heart failure; DM, diabetes mellitus; Serum creatinine; sodium; potassium; calcium; intubated; Mechanical ventilation use; Vasopressor use (1st 24 h); FiO_2_.

### 3.5 Identification of non-linear relationship

**FIGURE 2:**
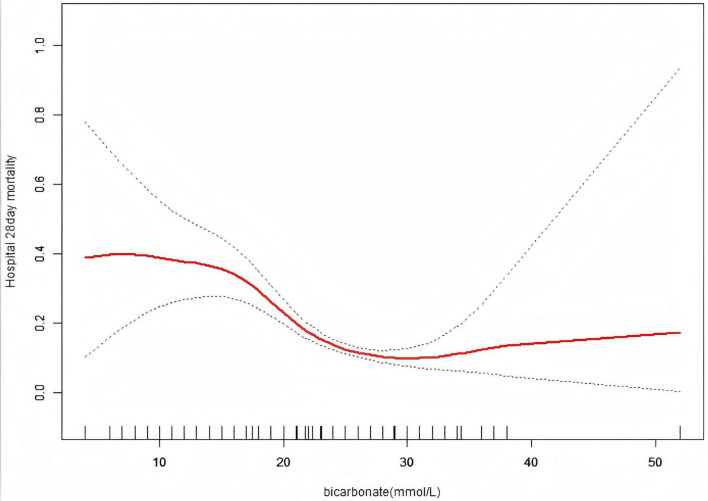
Association between serum bicarbonate levels and 28-day mortality in hospitalized patients. A threshold non-linear relationship was estimated using a generalized additive model (GAM). The solid red curve shows the smooth fitted association, and the flanking blue dashed lines indicate the 95% confidence interval. Rug marks on the x-axis indicate the distribution of serum bicarbonate values. The model was adjusted for age, gender, ethnicity; BMI, body mass index; WBC, white blood cell count; SOFA score, Sequential Organ Failure Assessment; COPD, chronic obstructive pulmonary disease; CHF, congestive heart failure, DM, diabetes mellitus; serum creatinine, sodium, potassium, calcium, intubation, mechanical ventilation use, Vasopressor use (1st 24 h); FiO_2_.

A nonlinear dose–response relationship was detected between serum bicarbonate levels and 28-day mortality (Figure 2). When serum bicarbonate was below 30 mmol/L, each 1 mmol/L increase was associated with a 11% reduction in mortality risk (OR = 0.89, 95% CI: 0.86–0.92, *p* < 0.0001). However, levels above 30 mmol/L were not significantly associated with changes in mortality risk (OR = 1.11, 95% CI: 0.97–1.28, *p* = 0.1278). A generalized additive model corroborated this nonlinear pattern. A likelihood ratio test comparing the linear and piecewise linear models yielded *p* < 0.001, indicating that the piecewise model provided a superior fit to the data (Table 4). The sensitivity analysis results were consistent with the main analysis; Supplementary analyses yielded similar results even after accounting for the impact of missing data (Supplementary Figure 1 and Supplementary Table 1); moreover, time-dependent analyses were conducted, and their outcomes were generally consistent with the main findings (Supplementary Figure 2 and Supplementary Table 2).

**TABLE 4:** Threshold effect analysis of serum bicarbonate and 28-day mortality.

Models	OR (95%CI)	*P*-value
**Model I**			
One line effect	0.90 (0.87, 0.93)	<0.0001
**Model II**			
Turning point^(K)^	30	
Albumin ratio < K	0.89 (0.86, 0.92)	<0.0001
Albumin ratio > K	1.11 (0.97, 1.28)	0.1278
*P-*value for LRT test*		0.014

Data were presented as OR (95% CI) *P*-value; Model I, linear analysis; Model II, non-linear analysis. Adjusted for age (years), gender, Ethnicity; BMI, body mass index; WBC, white blood cell; Serum creatinine, SOFA score, Sequential Organ Failure Assessment; COPD, chronic obstructive pulmonary disease; CHF, congestive heart failure; DM, diabetes mellitus; sodium; potassium; calcium; intubated; Mechanical ventilation use; Vasopressor use (1st 24 h); FiO_2_. CI, confidence interval; OR, odds ratio; LRT, logarithm likelihood ratio test.

*P < 0.05 indicates that model II is significantly different from Model I.

## 4 Discussion

This study identified a significant inverse association between serum bicarbonate levels and 28-day mortality in dialysis patients. The key finding is a non-linear relationship, whereby serum bicarbonate levels below 30 mmol/L were associated with a 11% reduction in mortality risk for each 1 mmol/L increase (adjusted OR = 0.89, 95% CI: 0.86–0.92, *p* < 0.0001). In contrast, bicarbonate levels above 30 mmol/L did not show a statistically significant association with mortality (adjusted OR = 1.11, 95% CI: 0.97–1.28, *p* = 0.1278). To our knowledge, this is the first study to comprehensively describe a non-linear association between serum bicarbonate levels and short-term mortality in this population. These findings warrant further validation.

Previous studies have consistently shown that lower serum bicarbonate levels are associated with increased mortality and adverse prognosis (14–16). In stable maintenance hemodialysis populations, prior studies have reported a U-shaped association between serum bicarbonate levels and mortality, whereby both lower and higher bicarbonate concentrations are associated with increased risk (17). For example, a retrospective cohort study of 5,835 patients with acute kidney injury (AKI) reported that bicarbonate concentrations below 22 mmol/L were linked to higher 14-day mortality and a greater risk of AKI progression. Similarly, Lombardi et al. demonstrated a U-shaped relationship between bicarbonate levels and mortality in a cohort of 1,485 elderly patients with advanced chronic kidney disease (CKD), with a statistically significant trend (*p* = 0.03) (18). These studies suggest that serum bicarbonate levels can serve as important prognostic markers in patients with kidney dysfunction, consistent with the findings of our analysis.

A retrospective study based on the MIMIC-IV database (*n* = 450) found a significant negative association between serum bicarbonate levels and 28-day mortality (19). Higher bicarbonate levels were associated with lower 28-day mortality, and this association remained significant after multivariable adjustment (adjusted HR = 0.94, 95% CI 0.89–0.99, *p* = 0.028) (19). Overall, each 1 mmol/L increase in bicarbonate corresponded to an approximately 10% reduction in mortality risk (HR = 0.90, 95% CI 0.85–0.95, *p* < 0.001) (19). Moreover, an “L-shaped” nonlinear relationship was observed, with an inflection point around 27 mmol/L; below this threshold, each 1 mmol/L increase was associated with about a 14% reduction in risk (HR = 0.86, 95% CI 0.79–0.94, *p* < 0.001) (19). In comparison, our study reported a 28-day mortality rate of 10.30% and identified a slightly higher inflection point at 30 mmol/L. Another retrospective cohort study, including 521 participants, found a negative correlation between serum bicarbonate levels and 30-day mortality (HR = 0.93, 95% CI: 0.88–0.98, *p* = 0.004) (20). However, that study did not clarify whether patients with non-traumatic subarachnoid hemorrhage received dialysis during hospitalization. In contrast, our study specifically examined short-term (28-day) mortality among dialysis patients and incorporated additional covariates. These differences suggest that responses to serum bicarbonate level variations may differ across clinical populations. Chang et al. conducted a prospective study on 441 peritoneal dialysis patients between 2000 and 2005, evaluating the association between serum bicarbonate levels and mortality (21). They found that patients with bicarbonate levels below 22 mmol/L and those between 22 and 24 mmol/L had 13.10- and 2.13-fold higher mortality risks, respectively, compared with the 24-26 mmol/L group. Moreover, each 1 mmol/L increase in bicarbonate was associated with a 17% reduction in mortality risk (HR = 0.83, 95% CI: 0.76–0.91, *p* < 0.001) (21). While our findings are generally consistent with this study, there are key differences. In our investigation, when serum bicarbonate levels were below 30 mmol/L, each 1 mmol/L increase was associated with a 11% reduction in mortality risk (adjusted OR = 0.89, 95% CI: 0.86–0.92, *p* < 0.0001). However, no significant association was found for serum bicarbonate levels above 30 mmol/L. Importantly, our ICU cohort is pathophysiologically distinct from stable maintenance hemodialysis populations: it likely includes both patients with acute kidney injury initiating dialysis and those with end-stage kidney disease on chronic maintenance therapy, each characterized by different acid–base targets, buffering capacities, and illness severities. Additionally, our study utilized the eICU database and focused specifically on dialysis patients, whereas the previous study targeted peritoneal dialysis patients. The two studies also differed in the covariates included. Our analysis adjusted for intensive care unit-specific parameters such as SOFA scores, electrolyte concentrations, intubation status, and mechanical ventilation, while the prior study emphasized demographic characteristics and comorbidities. Furthermore, the follow-up periods and analytical methods differed. Chang et al. reported a follow-up of 34.8 months, focusing on chronic mortality risks, while our study addressed short-term mortality. We employed generalized additive models (GAM), logistic regression, and threshold effect analysis, whereas the prior study used Cox regression. These methodological differences may contribute to varying interpretations, particularly regarding nonlinear patterns and threshold effects.

Serum bicarbonate plays a critical role in the regulation of body fluid balance, acid–base homeostasis, and several essential biological functions. Disruptions in this equilibrium are strongly associated with poor prognosis in critically ill patients (20, 22, 23). Low serum bicarbonate levels indicate the presence of metabolic acidosis, a condition linked to increased mortality through multiple complications, including cardiac dysfunction, hypotension, and heightened susceptibility to infection (24–26). Low bicarbonate levels below 22 mmol/L have been shown to directly exacerbate muscle atrophy and hypoalbuminemia by enhancing ubiquitin-proteasome activity and stimulating glucocorticoid-mediated protein degradation, thereby contributing to poor outcomes in these patients (27, 28). Moreover, inadequate serum bicarbonate impairs calcium-phosphorus homeostasis, which promotes osteogenic transformation of vascular smooth muscle cells and endothelial dysfunction. These processes accelerate atherosclerosis and increase the risk of cardiac arrhythmias (29–31). Recent studies have confirmed a significant association between low serum bicarbonate levels and elevated coronary artery calcification scores in hemodialysis patients (32, 33).

Bicarbonate deficiency also triggers overactivation of proinflammatory cytokines such as interleukin-6 (IL-6) and tumor necrosis factor-alpha (TNF-α) via the NF-κB signaling pathway, while concurrently inhibiting anti-inflammatory responses mediated by the Nrf2-Keap1 pathway. This dual effect is closely linked to an increased risk of 28-day mortality (34–36). Additionally, oxidative stress resulting from excessive mitochondrial reactive oxygen species (ROS) production and impaired antioxidant defense systems (such as reduced superoxide dismutase activity) further contributes to lipid peroxidation, DNA damage, and multi-organ failure in the presence of inflammation (37–40). Therefore, serum bicarbonate levels may affect mortality risk through multiple biological mechanisms that influence cardiovascular function and systemic metabolic regulation. In dialysis patients with significantly reduced serum bicarbonate levels, stringent monitoring is essential to closely monitor serum calcium levels, inflammatory cytokines, oxidative stress markers, and cardiac function. Early and targeted therapeutic interventions may be crucial to improving clinical outcomes.

Patients with serum bicarbonate levels < 22 mmol/L exhibited the highest mortality (14). Low bicarbonate is consistently associated with protein–energy wasting and systemic inflammation (41). Mechanistically, acidosis activates proteolytic pathways and pro-inflammatory signaling (e.g., NF-κB) while impairing antioxidative defenses, thereby promoting muscle catabolism, hypoalbuminemia, and immune dysregulation (7, 41). Conversely, higher serum bicarbonate, particularly when reflecting persistent metabolic alkalosis or aggressive dialysate bicarbonate prescriptions, has been linked to accelerated vascular calcification and increased mortality among maintenance hemodialysis patients (7). A more alkaline milieu can favor calcium–phosphate crystallization, facilitate osteogenic transdifferentiation of vascular smooth muscle cells, and disrupt endogenous calcification inhibitors such as matrix Gla protein and fetuin-A, providing biological plausibility for these associations (42, 43). Notably, specific serum bicarbonate thresholds associated with harm vary across studies, and the optimal target remains to be defined in prospective research.

This study possesses several notable methodological strengths. First, the analysis was based on a large, multicenter cohort drawn from the eICU database, encompassing detailed clinical data from more than 200,000 patients across 208 hospitals in the United States. Second, we identified a non-linear relationship between serum bicarbonate levels and 28-day mortality, and determined a threshold effect with an inflection point at 30 mmol/L. This threshold may provide a clinically meaningful target for early intervention in high-risk patients. Finally, our statistical models accounted for a broad range of potential confounding factors, including demographic variables, laboratory parameters, comorbid conditions, and critical care interventions. The inclusion of established prognostic indicators such as the SOFA score and serum electrolyte levels further strengthened the robustness and clinical applicability of our findings.

### 4.1 Study limitations

Several potential limitations of this study should be considered. First, due to the retrospective observational design, causal inferences cannot be drawn. Although multivariable analyses adjusted for established prognostic factors, such as invasive mechanical ventilation, endotracheal intubation, disease severity (SOFA score), vasopressor dose, and FiO_2_, residual confounding from unmeasured variables may persist. Therefore, the findings should be interpreted with caution. Future studies should leverage datasets with detailed information on dialysis modalities to better account for these clinically meaningful exposures. Second, the dataset contained missing values for certain covariates. We applied multiple imputation using established statistical techniques to minimize bias; however, the impact of missing data cannot be entirely excluded. Third, the study focused exclusively on short-term outcomes, specifically 28-day mortality, without evaluating long-term clinical endpoints. Additionally, the eICU database does not reliably distinguish dialysis modalities (e.g., hemodialysis, continuous renal replacement therapy, or peritoneal dialysis). As a result, we were unable to stratify outcomes by modality or evaluate modality-specific effects, which may introduce unaccounted heterogeneity and limit causal interpretation. Future work with datasets that capture dialysis modality, dose, and timing will be necessary to verify whether the observed associations differ by modality. Fourth, the eICU database does not reliably capture the necessary information to distinguish AKI from ESRD or to enable robust stratification by ICD codes at scale in this cohort. As a result, stratification by AKI versus ESRD was not feasible, which may introduce residual misclassification and unaccounted heterogeneity, potentially limiting causal interpretation. Future research leveraging datasets with granular dialysis indication, modality, and prescription details is needed to delineate phenotype-specific effects. Lastly, as an observational analysis, the study can only demonstrate associations between serum bicarbonate levels and mortality. It does not establish a direct causal link. Lastly, the generalizability of the findings may be constrained, as the cohort included only hospitalized patients aged 18 years or older who received dialysis. Therefore, these results may not apply to outpatient or non-dialysis populations.

## 5 Conclusion

In this multicenter retrospective cohort study, 4,979 patients receiving dialysis, a non-linear association was identified between serum bicarbonate levels and 28-day in-hospital mortality. The analysis demonstrated that lower serum bicarbonate levels were significantly associated with a higher risk of death, with a distinct inflection point observed around 30 mmol/L. These findings suggest that serum bicarbonate may serve as an important prognostic indicator in critically ill patients undergoing dialysis. Future prospective studies are necessary to validate these findings and to assess whether targeted management of serum bicarbonate levels could improve short-term outcomes in various patient populations.

## Data Availability

Publicly available datasets were analyzed in this study. This data can be found at: https://physionet.org/content/eicu-crd/2.0/, DOI: 10.13026/C2WM1R, PhysioNet-eICU Collaborative Research Database (eICU-CRD).
