# Supplementary material for: Association between serum bicarbonate levels and 28-day in-hospital mortality in dialysis patients: a multicenter retrospective cohort study based on the eICU Collaborative Research Database
Source: Front Med (Lausanne). 2025 Nov 12;12:1607191. doi: 10.3389/fmed.2025.1607191 (PMC12647071; doi:10.3389/fmed.2025.1607191)
Supplement: Supplementary file 1 [file Supplementary_file_1.doc]

**Supplementary Table 1**

| **Threshold effect analysis of serum bicarbonate and 28-day mortality** | | |
| --- | --- | --- |
|  |  |  |
| **Models** | **OR (95%CI)** | ***P*** value |
| Model I | | |
| One line effect | 0.91 (0.89, 0.93) | <0.0001 |
| Model II | | |
| Turning point (K) | 26.9 | |
| Lactate < K | 0.89 (0.87, 0.92) | <0.0001 |
| Lactate ＞K | 1.00 (0.93, 1.08) | 0.9614 |
| P value for LRT test* |  | 0.015 |
| Data were presented as OR (95% CI) P value; Model I, linear analysis; Model II, non-linear analysis. Adjusted for age (years), gender, Ethnicity; *BMI*, body mass index; *WBC*, white blood cell; Serum creatinine, *SOFA* score, Sequential Organ Failure Assessment; *COPD*, chronic obstructive pulmonary disease; *CHF*, congestive heart failure; *DM*, diabetes mellitus; sodium; potassium; calcium; intubated; Mechanical ventilation use; Vasopressor use; FiO2. *CI*, confidence interval; *OR*, odds ratio; *LRT*, logarithm likelihood ratio test. * *P*<0.05 indicates that model II is significantly different from Model I. | | |

**Supplementary Figure 1**

**Title: Association between serum bicarbonate levels and 28-day mortality in hospitalized patients.**


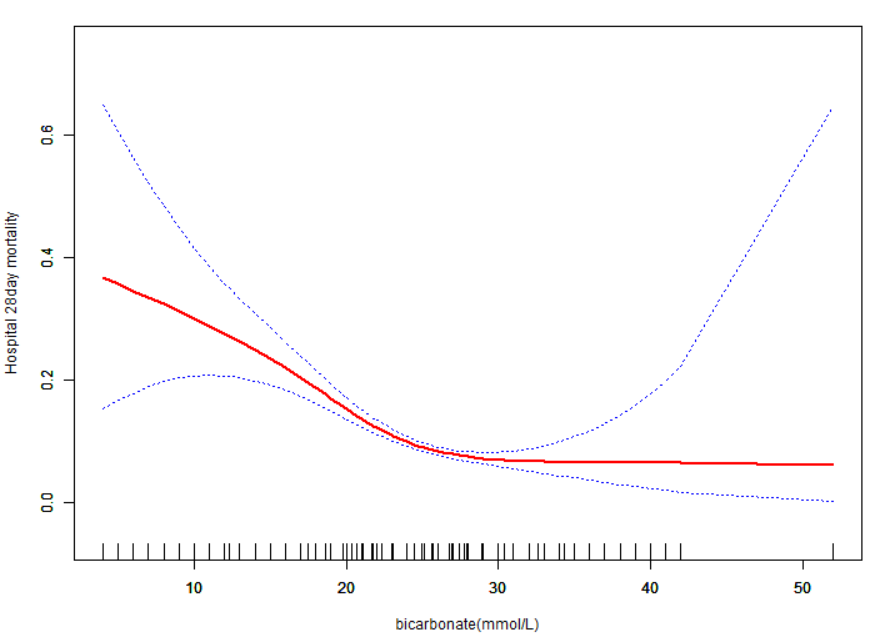


Note: A threshold non-linear relationship was estimated using a generalized additive model (GAM). The solid red curve shows the smooth fitted association, and the flanking blue dashed lines indicate the 95% confidence interval. Rug marks on the x-axis indicate the distribution of serum bicarbonate values. The model was adjusted for age, gender, ethnicity; BMI, body mass index; WBC, white blood cell count; SOFA score, Sequential Organ Failure Assessment; COPD, chronic obstructive pulmonary disease; CHF, congestive heart failure, DM, diabetes mellitus; serum creatinine, sodium, potassium, calcium, intubation, mechanical ventilation use, Vasopressor use, FiO2 .

**Supplementary Table 2:**

| **Threshold effect analysis of serum bicarbonate and 28-day mortality** | | |
| --- | --- | --- |
|  |  |  |
| **Models** | **OR (95%CI)** | ***P*** value |
| Model I | | |
| One line effect | 0.92 (0.90, 0.95) | <0.0001 |
| Model II | | |
| Turning point (K) | 30 | |
| Lactate < K | 0.92 (0.89, 0.94) | <0.0001 |
| Lactate ＞K | 1.09 (0.97, 1.22) | 0.1565 |
| P value for LRT test* |  | 0.037 |
| Data were presented as OR (95% CI) P value; Model I, linear analysis; Model II, non-linear analysis. Adjusted for age (years), gender, Ethnicity; *BMI*, body mass index; *WBC*, white blood cell; Serum creatinine, *SOFA* score, Sequential Organ Failure Assessment; *COPD*, chronic obstructive pulmonary disease; *CHF*, congestive heart failure; *DM*, diabetes mellitus; sodium; potassium; calcium; intubated; Mechanical ventilation use; Vasopressor use; FiO2. *CI*, confidence interval; *OR*, odds ratio; *LRT*, logarithm likelihood ratio test. * *P*<0.05 indicates that model II is significantly different from Model I. | | |

**Supplementary Figure 2**

**Title: The association between serum bicarbonate levels and 28-day mortality in hospitalized patients**


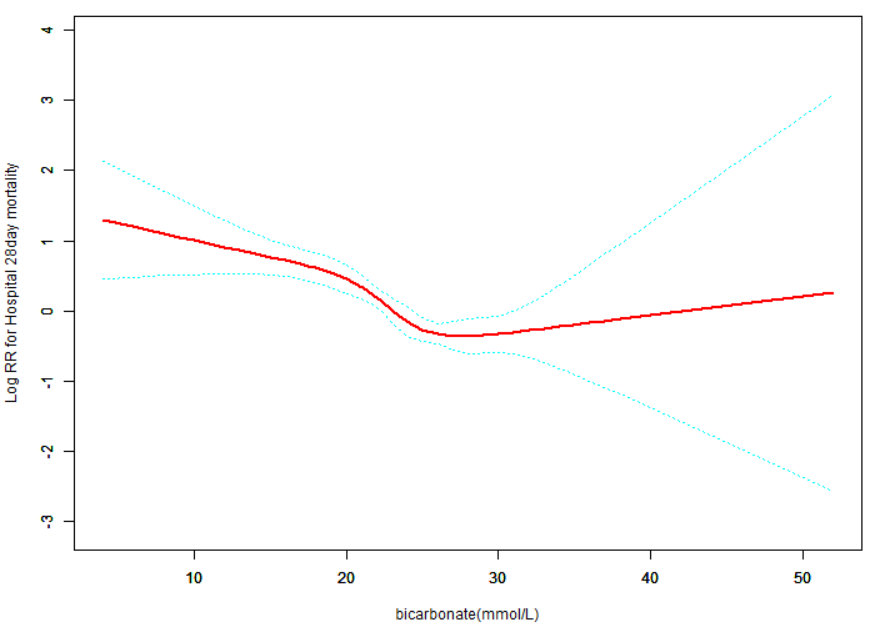


Note:A threshold non-linear relationship was estimated using a generalized additive model (GAM). The solid red curve shows the smooth fitted association, and the flanking blue dashed lines indicate the 95% confidence interval. Rug marks on the x-axis indicate the distribution of serum bicarbonate values. The model was adjusted for age, gender, ethnicity, *BMI*, body mass index; *WBC*, white blood cell count; *SOFA* score, Sequential Organ Failure Assessment; *COPD*, chronic obstructive pulmonary disease; *CHF*,congestive heart failure; *DM*, diabetes mellitus, serum creatinine, sodium, potassium, calcium, intubation, and mechanical ventilation use; Vasopressor use; FiO2.
